# The pp24 phosphoprotein of Mason-Pfizer monkey virus contributes to viral genome packaging

**DOI:** 10.1186/1742-4690-2-68

**Published:** 2005-11-07

**Authors:** Christopher R Bohl, Shanna M Brown, Robert A Weldon

**Affiliations:** 1 School of Biological Sciences and the Nebraska Center for Virology, University of Nebraska, Lincoln, 68588, USA

## Abstract

**Background:**

The Gag protein of Mason-Pfizer monkey virus, a betaretrovirus, contains a phosphoprotein that is cleaved into the Np24 protein and the phosphoprotein pp16/18 during virus maturation. Previous studies by Yasuda and Hunter (J. Virology. 1998. 72:4095–4103) have demonstrated that pp16/18 contains a viral late domain required for budding and that the Np24 protein plays a role during the virus life cycle since deletion of this N-terminal domain blocked virus replication. The function of the Np24 domain, however, is not known.

**Results:**

Here we identify a region of basic residues (KKPKR) within the Np24 domain that is highly conserved among the phosphoproteins of various betaretroviruses. We show that this KKPKR motif is required for virus replication yet dispensable for procapsid assembly, membrane targeting, budding and release, particle maturation, or viral glycoprotein packaging. Additional experiments indicated that deletion of this motif reduced viral RNA packaging 6–8 fold and affected the transient association of Gag with nuclear pores.

**Conclusion:**

These results demonstrate that the Np24 domain plays an important role in RNA packaging and is in agreement with evidence that suggests that correct intracellular targeting of Gag to the nuclear compartment is an fundamental step in the retroviral life cycle.

## Introduction

Viruses of the *Betaretroviruses *genus, formerly known as D- and B-type retroviruses, assemble their capsids in the cytoplasm of infected cells instead of at the plasma membrane like most retroviruses. The B-type viruses contain prominent surface glycoproteins and spherical, eccentric capsids and include mouse mammary tumor virus (MMTV) and exogenous and endogenous MMTV-like retroviruses in mice and humans [1-3]. D-type viruses have less dense surface spikes and contain cylindrical capsids. Exogenous and endogenous D-type viruses infect in a variety of mammalian hosts including Old World monkeys (Mason-Pfizer monkey virus [M-PMV], simian retrovirus 1 [SRV-1], [SRV-2] and simian endogenous retrovirus) [4-6], New World monkeys (squirrel monkey retrovirus [SMRV]) [7], sheep and goats (Jaagsiekte sheep retrovirus and enzootic nasal tumor virus respectively) [8-10]. D-type virus sequences have also been detected in humans, the Australian common brushtail possum and mice (*Trichosurus vulpecula *endogenous retrovirus D, rabbit endogenous virus H, and MusD, respectively) [11-13].

M-PMV, the prototypical D-type virus, was first isolated from a mammary adenocarcinoma of a female Rhesus monkey [14]. Although M-PMV was originally suspected to be an oncogenic virus, it was later found to induce a sever "wasting" and immunodeficiency syndrome distinct from that caused by immunosuppressive lentiviruses [15]. SRV-1 and SRV-2 are related to, yet serotypically distinct from, M-PMV and were isolated from primates suffering diseases similar to that caused by M-PMV [16,17].

M-PMV, the most thoroughly understood of the D-type betaretroviruses, contains four genes (5'-*gag-pro-pol-env*). As with other retroviruses, its Gag protein, Pr78, serves multiple functions during the viral life cycle, including virus assembly, virion maturation and early post-entry steps in virus replication [18]. Multiple studies have shown that Pr78 has the innate ability to assemble into immature capsids or procapsids in the cytoplasm, recognize and package the viral RNAs and glycoproteins and facilitate budding from the plasma membrane. During viral budding or shortly thereafter, Pr78 is cleaved by the viral protease to yield the mature virion associated proteins: matrix MA (p10), the phosphoprotein pp24, p12, capsid (CA or p27), nucleocapsid (NC or p14) and p4. These mature Gag-cleavage products then play roles during the early stages of the viral life cycle where they may help facilitate uncoating, reverse transcription and nuclear entry of the viral DNA. The regions and modifications of Pr78 required for these events have been partially identified.

Upon translation, Pr78 is targeted to a pericentriolar region of the cytoplasm in close proximity to the nuclear membrane where it assembles into spherical, procapsids [19]. The signal within Pr78 responsible for this pericentriolar targeting (the cytoplasmic targeting/retention signal or CTRS) is located within an 18 amino acid sequence of the matrix domain (MA). This motif is dominant over the bipartite myristylation and lysine/arginine-rich bipartite membrane targeting signals that is also located within the MA domain. Insertion of the CTRS into the analogous region of the MLV Gag protein, which normally assembles at the plasma membrane, results in intracytoplasmic assembly of MLV Gag. Secondly, substitution of an arginine within the CTRS of M-PMV Gag to a tryptophan (R55W) destroys the dominant CTRS function resulting in capsid assembly at the plasma membrane [20].

Other regions of Pr78 are also essential for procapsid assembly. Residues within the MA, yet separate from the CTRS, and the CA domains are required for assembly [20-23]. Likewise, the p12 domain with in Pr78 provides an internal scaffolding that together with the cononical I domain, which is located near the CA-NC junction, function to promote Gag-Gag interactions during capsid [24,25]. Assembly of the spherical capsid also requires interactions between the viral RNAs (vRNA) and Gag proteins [26,27]. Thus, the vRNA must also be present at the assembly site to provide this additional nucleation or scaffolding function. Although Gag-vRNA interaction occurs primarily though interaction in the NC domain, other regions of Gag appear to be important by targeting Gag to the site of vRNA packaging and by imparting correct structural information upon Gag [22,28-32].

Following assembly, the procapsids are transported to the plasma membrane from which they bud. Both the myristyic acid modification of Pr78 and specific amino acids within the MA domain play critical roles in plasma membrane targeting [20,21]. Moreover, Sfakianos and Hunter have shown that the M-PMV Env glycoproteins and Rab11-positive recycling endosomes play critical roles in transporting the preassembled procapsids from the pericentriolar assembly site to the plasma membrane [33]. Upon arrival of the procapsids at the plasma membrane, a proline-rich PPPYX_4_PSAP motif located near the carboxy-terminus of the Gag phosphoprotein, pp24, provides the late budding domain (L), which facilitates viral budding [34,35].

Upon release, nascent immature particles undergo a maturation process to acquire infectivity. During this process, Pr78 is cleaved by the viral protease to yield the seven proteins: MA, pp24, p12, CA, NC, and p4. The phosphoprotein conserved among M-PMV, SRV-1, SRV-2, SERV, and MMTV yet its function is only partially known. During M-PMV maturation, pp24 is further cleaved into two proteins; the C-terminal pp16 protein and the N-terminal Np24 protein. Both are required for virus replication. Yasuda and Hunter demonstrated that the pp16 domain contains the late budding motif and deletion of the Np24 domain completely blocked virus replication [35]. However, the function of Np24 was not determined. In this study, we examined the role of the Np24 domain during virus replication. We have identified a lysine-arginine rich motif within Np24 that is conserved among many betaretrovirus and is essential for infectivity. The results presented here show that the KKPKR motif in Np24 is not required for procapsid assembly, intracellular transport, budding or glycoprotein incorporation but plays a critical role in vRNA packaging.

## Results

### Deletion of the KR box in Np24 blocks virus replication

**Figure 1:**
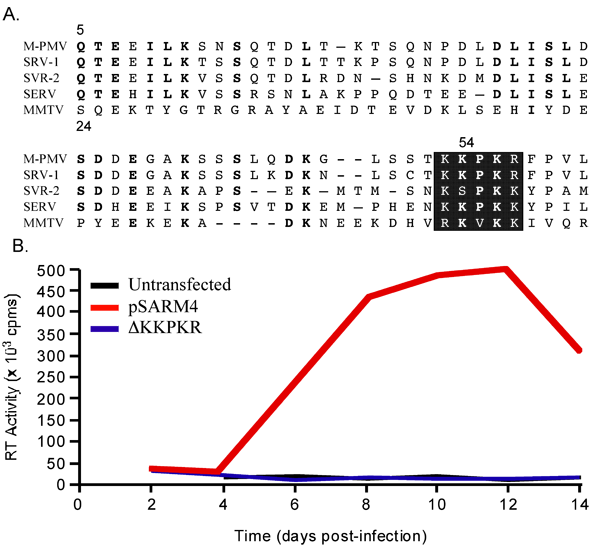
The highly conserved KR box within the Np24 protein of M-PMV is required for viral replication. (A) Amino acid alignment of phosphoproteins of betaretroviruses showing areas of sequence conservation in the N-terminus and C-terminus. All conserved residues are bolded and the highly conserved KR box is shadowed. Accession Numbers; M-PMV (P07567), SRV-1 (AAA47730), SRV-2 (P51516), MMTV (AAF3147) (B) Viral replication measured by virus spread assay. Culture media from COS-1 cells transfected with nothing (Black), wild-type M-PMV proviral DNA, pSARM4 (Red), or ΔKKPKR proviral DNA (Blue) were collected and assayed for RT activity. HOS cells were infected with equal amounts of virus (normalized by RT activity). Virus spread in HOS cells was measured by RT assays at 2, 4, 6, 8, 10, 12, and 14 days post infection.

While it was previously determined that the Np24 domain of Pr78^Gag ^is required for replication [35], the role of this protein plays during the virus life cycle is not known. To gain further insight into which regions(s) of Np24 might be important for replication, the Np24 protein sequence was aligned to the analogous phosphoproteins from different infectious betaretroviruses (Fig. 1A). As expected because of the close similarity between the simian *betaretroviruses *M-PMV and SRV-1 and the more distantly related SRV-2 and SERV, their phosphoprotein sequences are 82%, 62%, and 61% (respectively) similar to M-PMV Np24. The most notable similarities occur at the amino- and carboxy-terminal ends of theses phosphoproteins. While the amino-terminal sequences are not conserved in the phosphoproteins of MMTV and MMTV related betaretroviruses, the highly conserved cluster of positively charged amino acids, KKPKR, (the KR box) near the carboxy-terminal end is shared by these betaretroviruses. In M-PMV (and SRV-1), the KR box is located near the carboxy-terminal end of Np24. In MMTV, it is located in the same relative position of pp21. The conservation of the KR box within the phosphoproteins of these divergent viruses suggests that it may be essential to virus replication. To determine if this motif (KKPKR) in M-PMV serves an important role during the virus life cycle, PCR mutagenesis was used to delete the region encoding the KKPKR motif from the infectious clone pSARM4. The mutant, pΔKKPKR, and wild-type pSARM4 proviral DNAs were transfected into COS-1 cells. At 48 h post transfections the viruses produced from the transfected cells were harvested and assayed for RT activity. Wild-type M-PMV and ΔKKPKR-transfected cells produced equivalent amounts of virus particles (data not shown). Viral spread assays were then carried out to examine if the deletion of the KKPKR motif affected viral replication. For this, Hos cells were infected with equivalent amounts of wild-type and mutant virus particles, normalized by RT activities. The amounts of virus particles present in the supernatants of infected cells at 2, 4, 8, 10, and 12 days post-infection were determined by RT assays. While wild-type virus replicated in Hos cells, as indicated by the increasing amounts of RT activity in the culture medium over time, no detectable RT activity was observed in the supernatants of uninfected or ΔKKPKR-infected COS-1 cells even at 14 days post-infection (Fig. 1B). These data demonstrates that deletion of the KKPKR motif blocked virus replication.

### Assembly and release of ΔKKPKR mutant particles

**Figure 2:**
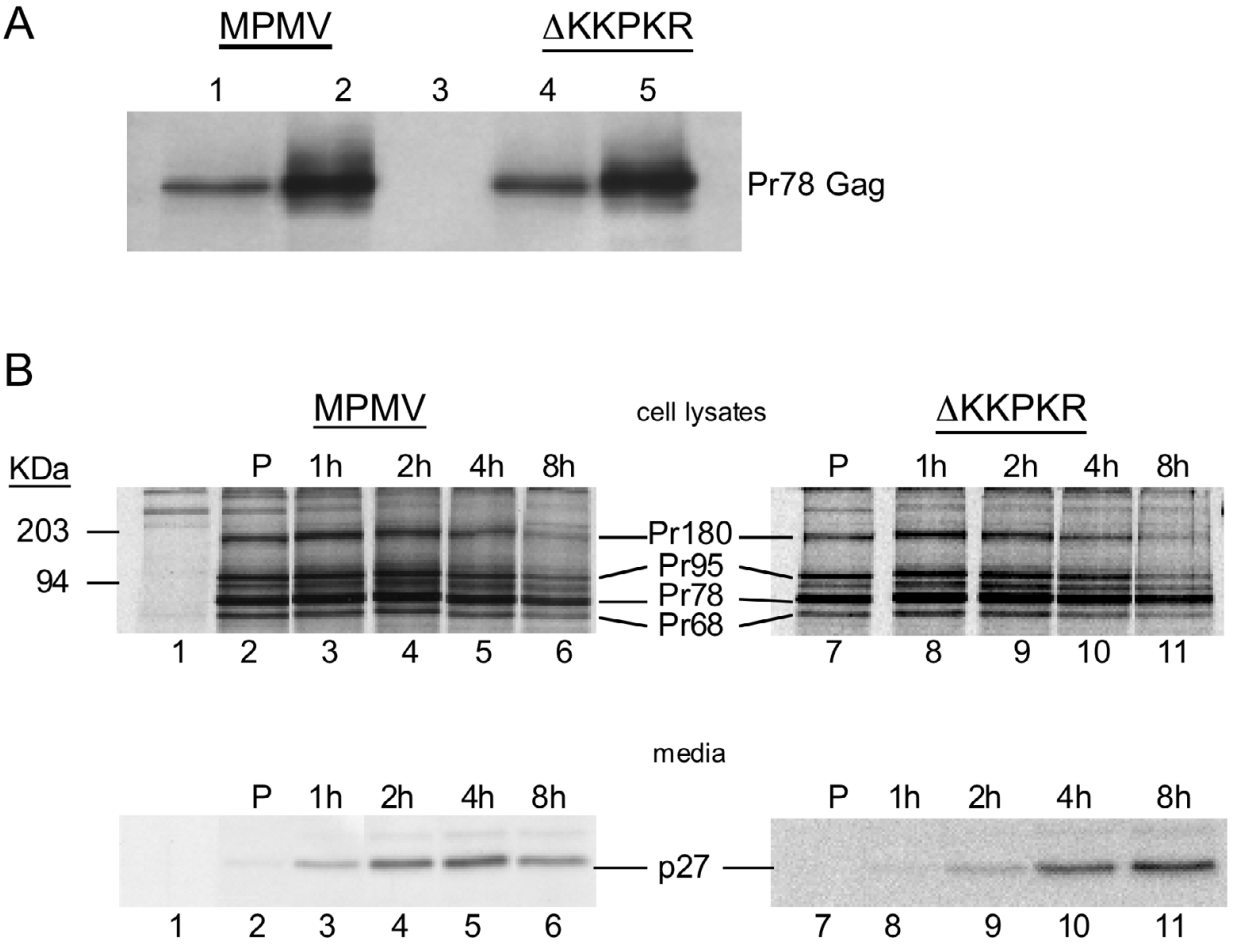
(A) Western Blot analysis of intracellular procapsid assembly using Gag fractionation techniques. 48 hrs post transfection, COS-1 cells were lysed with and fractionated over a 20% sucrose cushion to separate assembled procapsids from unassembled Gag proteins. Pr78 in the fractionated samples were detected by western blot using rabbit anti-Pr78 antibodies. Soluble wild-type Pr78 (lane 1); pelletable wild-type Pr78 (lane 2); untransfected (lane 3); soluble ΔKKPKR Pr78 (lane 4); pelletable ΔKKPKR Pr78 (lane 5). (B) Virus release kinetics. Transfected COS-1 cells pulsed labeled with [35S] methionine-cysteine for 30 minutes and chased for 0, 1, 2, 4, and 8 hours. Untransfected (lane 1); pSARM-4 (lanes 2–6); ΔKKRKR (lanes 7–11). Medium was collected and cells were lysed at the appropriate times with 1× Buffer A. Cellular lysates and medium were adjusted to 1× lysis buffer B. Viral proteins were immunoprecipitated from all samples using rabbit anti-Pr78 antibodies and separated by SDS-PAGE (12% acrylamyde) and detected by phospor-imaging.

Because the different morphogenic steps (procapsid assembly, cytoplasmic transport, membrane biding, and budding) are temporally separate for M-PMV, this virus provides an ideal opportunity to determine which if any of the late assembly steps might be affected by the deletion mutation. To determine if the replication-defective ΔKKPKR mutant could assemble intracellular procapsids, transfected cells were lysed in a TX-100 lysis buffer that does not disrupt assembled procapsids. The assembled procapsids were separated from the soluble unassembled Gag proteins in the cellular lysates by centrifugation through a 20% sucrose cushion. The pelleted proteins were solublized directly in protein loading buffer, separated by SDS-PAGE, and immunoblotted using anti-Pr78 antibodies. As expected, wild-type Gag (Pr78^WT^) was detected in both the soluble fraction (unassembled) and pelleted fractions (assembled procapsids) of the cellular lysates (Fig 2A. Lanes 1 and 2). The presence of the ΔKKPKR mutant Gag protein (Pr78^ΔKR^) in the soluble and pelletable fractions (Fig. 2A lanes 4 and 5) indicates that the mutant Gag proteins assembled into procapsids.

The rate of assembly and release of capsids from Pr78^WT ^and Pr78^ΔKR ^expressing cells were analyzed by pulse-chase analyses to determine if the deletion had caused defects in virus assembly and release. Transfected COS-1 cells were pulse labeled with [^35^S] methionine-cysteine for 30 min. and chased for 1, 2, 4, and 8 hours in complete growth medium. Cell associated and released-virus-associated proteins at each time point were analyzed by immunoprecipitation using rabbit anti-Pr78 antiserum (Fig. 2)

Similar levels of Gag (Pr78), Gag-Pro (Pr95), and Gag-Pro-Pol (Pr180) fusion proteins were synthesized during the pulse labeling by cells expressing wild-type M-PMV (Fig. 2B). The 68 kDa protein is a N-terminal truncated Gag protein that is expressed by initiation of translation at an internal methionine codon of *gag *at position 100 [24]. As expected, the intensities of these wild-type Gag proteins in the cell lysates decreased during the chase with a concomitant appearance of p27 (CA). During or shortly after virus release, Pr78^WT ^is processed by the virus-encoded protease into p10 (MA), Np24, pp16/18, p12, p27 (CA), p14 (NC), and p4. Thus the appearance of p27 in the culture medium indicates that virus particles were released and that Pr78^WT ^was being processed normally (Fig. 2B, lane 4). The other Gag cleavage products were not detected because they either do not contain methionines, or contain only a single methionine and thus were not detected.

**Figure 3:**
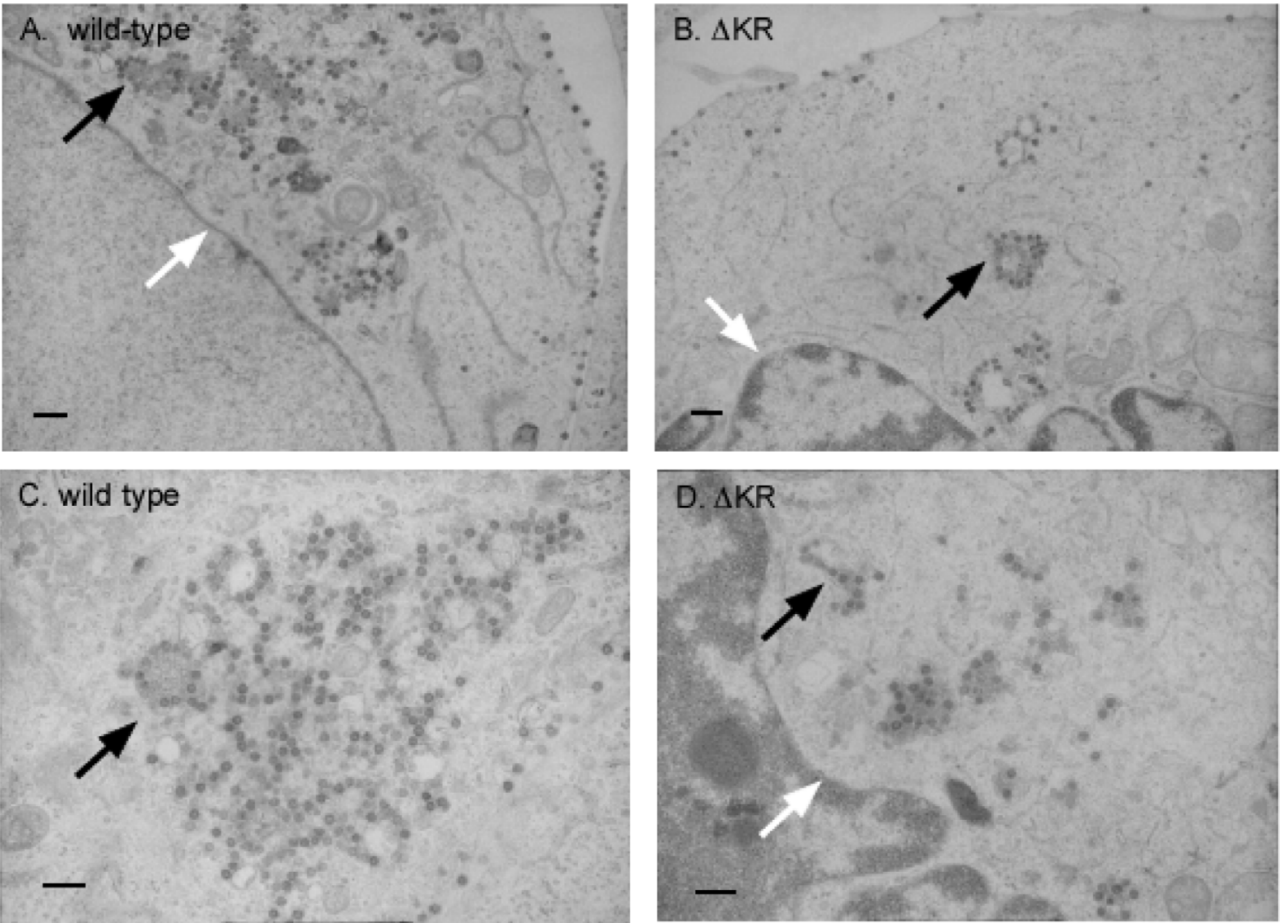
Intracellular procapsid morphology viewed by electron microscopy. COS-1 cells were transfected with pSARM-4 (A and C) or the ΔKKPKR mutant (B and D) proviral DNAs. Wild-type and mutant procapsids observed in close proximity to the nuclear membrane (white arrow) and throughout the cytoplasm near intracellular vesicles (black arrows). Bars approximately 500 nm.

In cells expressing the mutant virus, similar levels of cell-associated Gag precursors were observed in the pulse. Yasuda and Hunter [35] previously reported that deletion of the entire Np24 domain from Pr78 caused a rapid turn over of Pr78 in cells and thus decreased the amount of particles released from cells. It was, therefore suggested that the Np24 domain is important for Pr78 stability. However, deletion of just the KKPKR motif did not alter the intracellular stability of Pr78^ΔKR^. Instead, the intensity of Pr78^ΔKR ^decreased in the cell lysates in a manner similar to Pr78^WT^. This was accompanied by a slightly reduced rate of release of virus particles; Pr78^WT ^can first be detected in the medium after 1 h (Fig. 3B), while Pr78^ΔKR ^was first detected in the medium after 2 h (Fig. 3B). Thus, Pr78^ΔKR ^efficiently assembled into procapsids and released processed Gag (p27) in the culture medium with kinetics similar to Pr78^WT^.

### ΔKKPKR intracellular procapsids are indistinguishable from WT procapsids

The metabolic labeling and cell fractionation experiments provided biochemical evidence that the deletion of the KKPKR motif did not affect the ability of Gag to assemble into procapsids and be released from cells. Examining cells expressing Pr78^WT ^and Pr78^ΔKR ^by thin-section EM provided further evidence of normal assembly. Both produce intracellular, spherical procapsids (70–90 nm dia.) that have the characteristic, ring-shaped core typical of immature particles (Fig. 3). In addition, wild-type procapsids were found near the nuclear membrane, which others have shown to be the site of intracellular assembly [19] and near intracellular membranes (Fig. 3A and 3C). Interestingly, Sfakianos and Hunter have previously shown that Pr78^WT ^co-localizes with Rab11^+ ^recycling endosomes [33]. Whether the vesicles shown here are recycling endosomes is not yet known. Of note, we observed fewer assembling, or fully assembled procapsids near the nuclear membrane compared to wild-type (Fig. 3B and 3D) suggesting that at least part of the replication defect may be due to defect in intracellular targeting of newly synthesized Pr78^ΔKR ^proteins.

### ΔKKPKR Gag maturation and Envelope packaging

**Figure 4:**
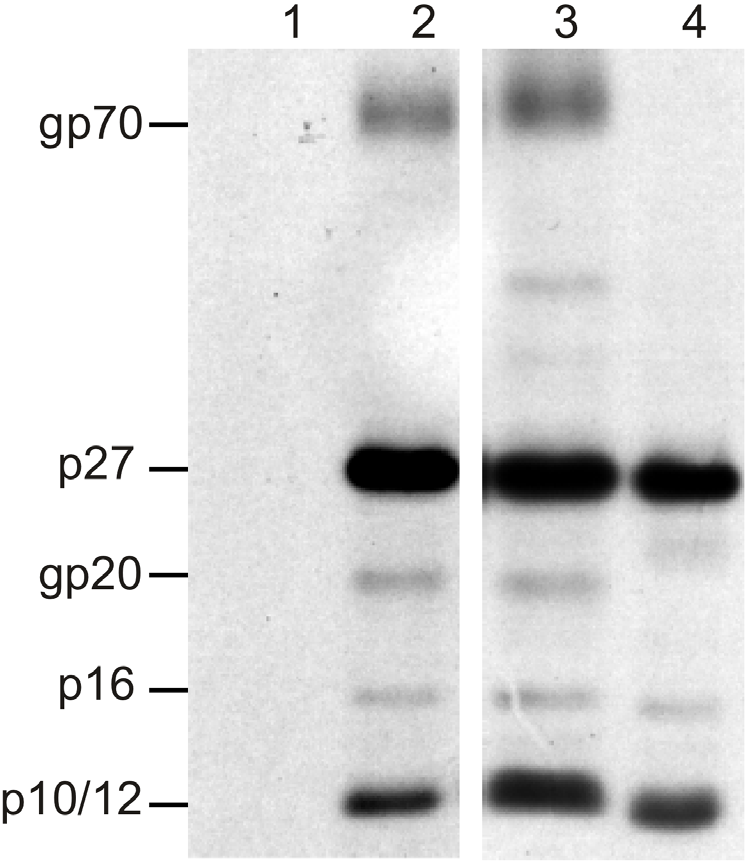
Glycoprotein incorporation and Gag processing. COS-1 cells were transfected with nothing (lane 1), pSAMR4 (lane 2), ΔKKPKR (lane 3), or pMT ΔE (lane 4) and then labeled overnight with [^3^H] leucine. Culture medium was filtered and viral particles were pelleted through a 20% sucrose cushion. The viral proteins present in the pellet were immunoprecipitated using goat anti-M-PMV antibodies. Positions of various viral proteins are indicated.

The cell fractionation and pulse-chase experiments combined with the EM analyses showed that the ΔKKPKR deletion mutant was released from cells as virus-like particles. Furthermore, the presence of reverse transcriptase activity and the CA (p27) protein in the culture medium of ΔKKPKR transfected cells suggest that the deletion did not affect PR-mediated processing of Gag, Gag-Pro or Gag-Pro-Pol (Fig. 1 and 2). The other Gag cleavage products (MA, p24/pp18, p12, NC, and p4) were not detected in the [^35^S] methinione labeling experiments because they do not contain a sufficient number of methionine residues for detection. Furthermore, these experiments could not determine if the viral glycoproteins, SU and TM (gp70 and gp20, respectively) were incorporated into the released particles because an anti-pr78-specific antibody used for the immunoprecipitations. It was therefore possible that the KR box deletion mutation either inhibited Env glycoprotein incorporation or precise Gag processing, thus blocking infectivity. To address this possibility, we looked for the presence of the Env glycoproteins and the other Gag cleavage products in released virions. To this end, COS-1 cells were transfected with pSARM4, pΔKKPKR or an Env-deletion mutant, pMT.ΔE and labeled overnight with [^3^H] leucine. Viral particles were pelleted through a 25% sucrose cushion, lysed, and immunoprecipitated using an anti-M-PMV antisera that recognizes the Gag cleavage products as well as gp70 and gp20. Figure 4 shows both wild-type and ΔKKPKR mutant particles contain the Env glycoproteins (gp70 and gp20) and the mature Gag cleavage proteins (MA [p10], pp16, p12, and CA [p27]). The NC (p14) and p4 cleavage products were not detected with the antiserum used. As expected particles released from pMT.ΔE transfected cells did not contain the gp70 or gp20 glycoproteins. These results demonstrate that the block to ΔKKPKR replication is not due to abnormal Pr78^ΔKR ^processing or an inability of the mutant particles to incorporate the viral glycoproteins.

### Genome packaging

**Figure 5:**
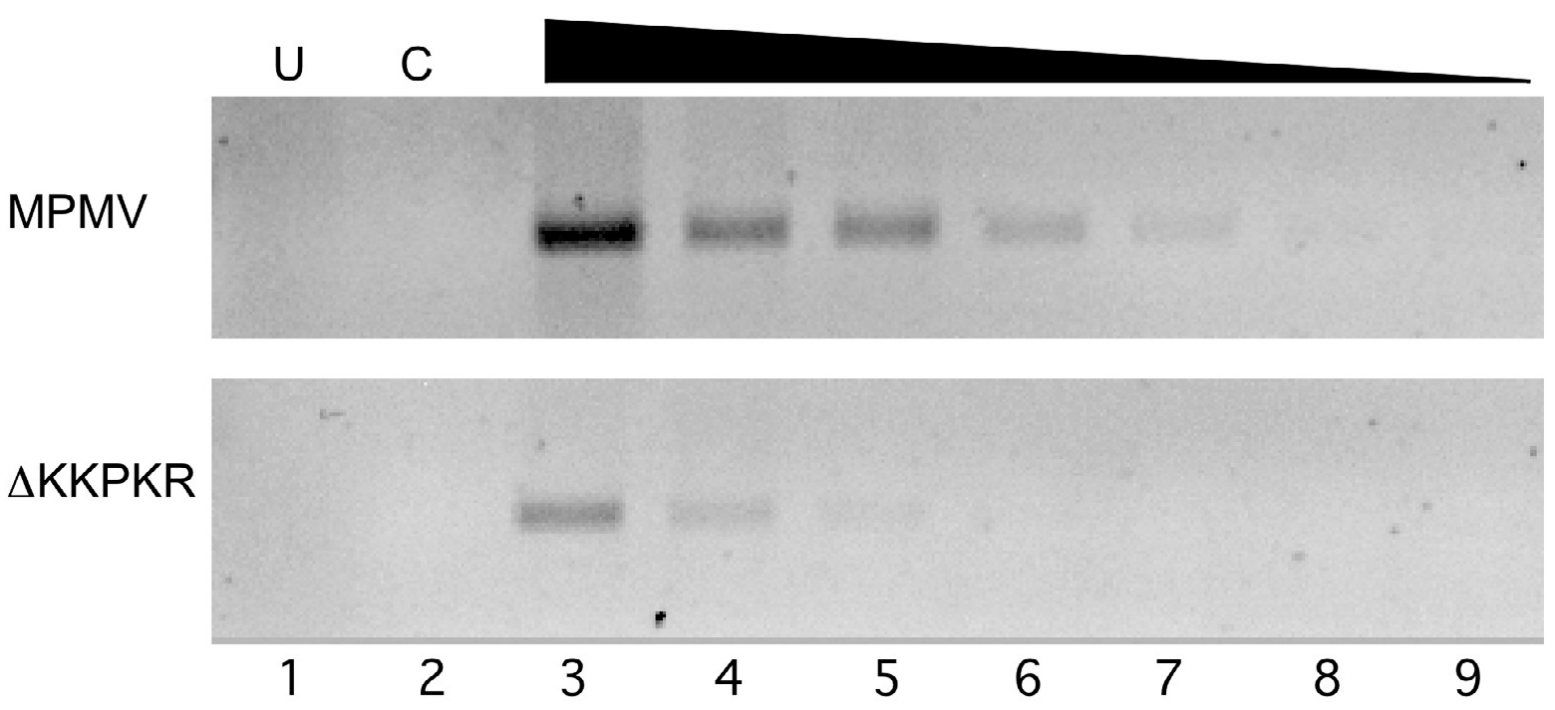
RT-PCR analysis of genome packaging in wild-type M-PMV and ΔKKPKR virions. Purified RNA from equivalent amounts of virus was diluted 1:1,000 (lane 3) followed by 2-fold serial dilutions to 1:96,000 (lane 9). First-strand cDNA synthesis was carried out using M-MLV RT and followed by PCR using oligos that amplify M-PMV CA sequences. Relative viral RNA packaging efficiencies were estimated by determining the end-point dilution in which viral PCR products could be detected by Ethidium bromide staining. U, untransfected (lane 1); C, RNA control – no reverse transcriptase added to RT-PCR reaction (lane 2).

Having demonstrated that the deletion of the KR box did not affect the packaging or processing of the *gag*-, *pol*-, and *env*-encoded viral proteins, semi-quantitative RT-PCR assays were utilized to address whether the deletion of the KR box altered packaging of genomic RNA into virions. Equivalent amounts of virus, normalized by p27 content from wild-type and mutant virions were pelleted through a 20% sucrose cushion and resuspended in PBS and the viral RNAs were extracted. Two-fold serial dilutions of viral RNAs were used for RT-PCR reactions using primers to amplify CA-coding sequences. The relative amounts of viral RNA that were packaged were estimated by determining the end-point dilution within which viral cDNAs could be detected by ethidium bromide staining. As shown in this representative experiment, the deletion of the KKPKR motif resulted in a 6–8 fold decrease in genome packaging, relative to wild-type (Fig. 5). Similar results were found using northern blot and dot-blot analyses of vRNAs using a riboprobe specific for M-PMV LTR sequences (data not shown). We concluded from these vRNA packaging assays that the deletion of the KR box significantly reduced the efficiency of vRNA packaging.

### Subcellular localization

Electron microscopic examination indicated that fewer numbers of ΔKKPKR procapsids were present near the nuclear membrane and suggested that the deletion of the KR box influences intracellular targeting of Pr78^ΔKR ^to this perinuclear site of assembly. This observation combined with the finding that Pr78^ΔKR ^packages significantly less vRNA into particles lead us to hypothesize that correct intracellular targeting and vRNA packaging are linked. This hypothesis is supported by previous studies with Rous sarcoma virus (RSV) and bovine leukemia virus (BLV) which demonstrated that basic residues in the regions of Gag proteins distant from the RNA binding motif within NC influences both Gag targeting and viral RNA packaging [29,30,32,36].

Studies with RSV have shown that its Gag proteins cycles through the nuclear compartment using a nonclassical nuclear targeting sequence within the MA domain and is exported out of the nucleus via the CRM-1 export pathway. Scheifele *et al*. have shown that treating the RSV Gag-expressing cells with the CRM-1 inhibitor leptomycin B (LMB) results in a dramatic accumulation of RSV Gag proteins within the nucleus. In addition, it has been shown that RSV MA mutants that are not targeted to the nuclear compartment are insensitive to LMB treatment and are released from cells as virus-like particles, yet are not infectious due to a defect in vRNA packaging. These results suggests that nuclear localization of RSV Gag and genome packaging are linked [29,36]. Likewise, Wang *et al*. have shown that basic residues within the MA domain of BLV are involved in vRNA packaging [30]. However, BLV Gag was not detected in the nucleus of cells treated with LMV. These results suggest that BLV Gag either does not enter the nucleus or that Gag does enter the nucleus but is exported by a CRM1-independent pathway.

**Figure 6:**
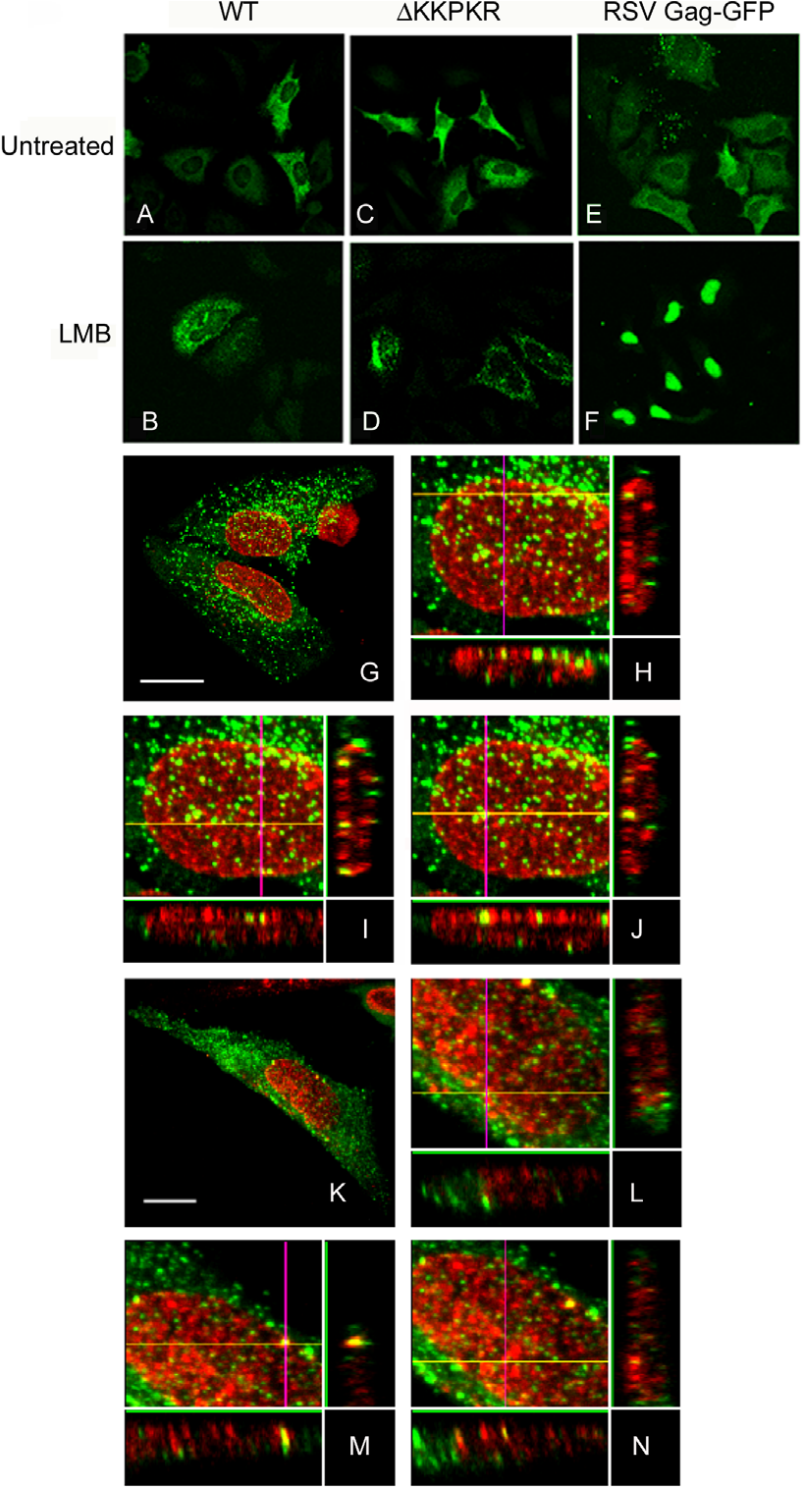
Subcellular localization of wild-type M-PMV Gag, ΔKKPKR Gag, and RSV Gag-GFP under steady state growth conditions or after treatment with LMB. HeLa cells were transfected with either pSARM-4, ΔKKPKR, or RSV Gag-GFP and left untreated or treated with LMB. The cells were fixed in methanol and the subcellular localizations of Gag were viewed by confocal microscopy using rabbit anti-Pr78 antibodies and Cy2 conjugated secondary antibodies. RSV Gag-GFP was directly visualized by fluorescence of the Gag-GFP fusion protein. Drug treatments: Wild-type M-PMV (untreated, panel 6A), wild-type MPMV (LMB treated, panel 6B) ΔKKPKR (untreated, panel 6C, ΔKKPKR (LMB treated, panel 6D), RSV Gag-GFP (untreated, panel 6E), and RSV Gag-GFP (LMB treated, panel 6F). Colocalization of wild-type M-PMV Gag, ΔKKPKR, and nuclear pores. Transfected HeLa cells were fixed with 4% paraformaldyhyde, and permiablized with 0.2% TX-100. Wild-type Gag (panels G-J), ΔKKPKR Gag (panels k-N), and nuclear pore localization were visualized by confocal microscopy using affinity purified anti-Pr78 and MAb414 antibodies, respectively, and counter stained with Cy2 anti-rabbit and Cy5 anti-mouse antibodies. 0.3 um Z-sections were stacked and orthogonal views through the cell were generated using Flowview imaging analysis software.

To further explore the intracellular trafficking of Pr78^WT ^and Pr78^ΔKR^, the steady-state intracellular locations of both were analyzed by confocal microscopy. Figure 6, which are representative z-sections of transfected COS-1 cells, shows that the highest concentration of both Pr78^WT ^and Pr78^ΔKR ^were routinely found throughout the cytoplasm. Interestingly, small amounts of Pr78^WT ^were also observed associated with the nuclear compartment. In contrast, nuclear staining of Pr78^ΔKR ^was only occasionally observed (Figure 6A and 6B, respectively). To examine if M-PMV Gag transiently traffics through the nuclear compartment in a CRM-1-dependent manner similar to RSV, we asked whether M-PMV Gag could be trapped within the nucleus by inhibiting the CRM-1 nuclear export pathway with LMB. As has been previously described [36], RSV Gag-GFP proteins were readily concentrated within the nucleus upon treatment with LMB (Fig. 6E–F). In contrast, LMB did not concentrate either Pr78^WT ^or Pr78^ΔKR ^in the nucleus (Fig. 6A–D).

Although these immunofluorescence experiments demonstrated that M-PMV does not utilize a CRM-1-dependent nuclear export pathway, it is possible that M-PMV Gag either enters the nucleus, and is exported in a CRM-1-independent manner, or Gag does not enter the nucleus but instead localizes to the nuclear pores. To distinguish between these two possibilities, we utilized confocal microscopy, an affinity-purified rabbit polyclonal anti-Pr78 antibody and a monoclonal antibody (MAb414) that recognizes conserved FG repeats of nuclear pore proteins [37] to examine whether Gag co-localizes with nuclear pores. Following confocal imaging of HeLa cells expressing either Pr78^WT ^or Pr78^ΔKR^, 0.3 um optical z-sections were stacked and orthogonal views through the nuclei were analyzed. In these cells, the nuclear pores were easily identifiable as punctate staining areas on the nuclear membrane. Moreover, Pr78^WT ^was not randomly dispersed within the nuclear compartment or around the nuclear membrane but rather concentrated in distinct foci in close proximity to nuclear pores (Fig. 6G–J). In contrast Pr78^ΔKR ^did not readily associate with nuclear pores (Fig. 6K–N), but instead localized mainly in the cytoplasm and as discrete foci adjacent to, but not associated with nuclear pores. However, we occasionally, but very infrequently, observed Pr78^ΔKR ^associating with nuclear pores.

## Discussion

The results of the studies described here show that a KKPKR sequence located near the carboxy-terminal end of the M-PMV Np24 domain of Gag plays a critical role during virus replication. Initial experiments showed that deletion of this motif did not inhibit the release of virions as demonstrated by the release of virion-associated RT activity into the culture medium of transfected cells. However, this mutant was unable to replicate, similar to what was observed when the entire Np24 domain was deleted [35]. While it is possible that the replication defect was due to the deletion inducing deleterious conformational changes in Pr78, several observations argue that this is unlikely. First, the mutant was capable of assembling into spherical procapsids with morphologies indistinguishable from wild-type. Second, these mutant procapsids, like wild-type, associated with intracellular membranes as has been described as a normal transport pathway for several retroviruses [33,38,39]. Finally, they packaged normal levels of the viral glycoproteins, gp70 and gp20, into the released virions. These results show that the deletion did not significantly alter the confirmation of Pr78 and they demonstrate that the basic residues in Np24 are not involved in targeting Gag to cellular membranes or in packaging the viral glycoproteins.

Further analyses showed that the deletion resulted in two assembly-related defects, vRNA packaging and intracellular targeting. Semi-quantitative PCR, northern blot and dot blot analyses routinely demonstrated that the mutant packaged 6–8 fold less vRNA than did wild-type. In vitro assembly assays have shown that assembly of RSV and HIV-spherical capsids requires nucleic acids [26,40]. In the absence of nucleic acids, Gag proteins assemble into sheets and tubes. Although we have not yet determined whether the spherical, cytoplasmic procapsids assembled from the ΔKKPKR mutant contained RNA (presumably mainly cellular RNAs), we would assume that the RNA content of those intracellular capsids is similar to that found in the released capsids.

Several explanations could account for the failure of this mutant to specifically package vRNAs. First, the Np24 domain may be directly involved in RNA packaging. While there is no evidence that the Np24 domain directly binds RNA or interacts with the NC protein in the virion, the presence of these basic residues may help facilitate NC-mediated vRNA packaging in a manner analogous to that hypothesized for the MA domain of bovine leukemia virus [30].

Another explanation for the defect in genome packaging, although unlikely, is that the deletion disrupted the viral RNA dimerization and/or packaging signals. While it has been proposed that dimer formation is required for RNA packaging, the relationship between dimer formation and packaging is still unclear. Nonetheless, for RSV, MLV, and HIV, the sequence elements involved in dimerization are included in the packaging signals located near the 5'-end of their respective *gag *genes. In addition, many in vivo studies have shown that mutations that disrupt RNA dimer formation also interfere with RNA packaging. For M-PMV, the RNA dimer initiation sequence (DIS) has not been precisely mapped. However, based on the mapping of the DIS within RNA packaging signals in other retroviruses (reviewed in [41]), the M-PMV DIS is also likely to be an integral part of the RNA packaging signal (Ψ). Because the ΔKKPKR deletion is more than 400 nucleotides down-stream of Ψ packaging signal [42], it is unlikely that the deletion mutation affected the cis-acting elements required for vRNA dimerization or packaging. We are currently determining if the few viral RNAs detected in the ΔKKPKR particles exist as dimmers and whether the mutant vRNA can be packaged in trans with wild-type Gag.

Previous studies on M-PMV suggested that the Np24 domain is required for Pr78 stability. Yasuda and Hunter [35] showed using pulse-chase experiments that deletion of the entire Np24 domain resulted in a Gag protein that when expressed in transfected cells, was more unstable than wild-type. The data presented here suggests that Np24, and perhaps more specifically the KKPKR motif, also plays an important role in intracellular targeting of Pr78. Based on the observations that relatively few ΔKKPKR mutant capsids were found in the perinuclear region of the cytoplasm, which has been shown to be the site of assembly, as well as the findings that wild-type Gag, but not the mutant, localized with the nuclear pores, we hypothesize that the KKPKR motif plays a role in targeting Gag to the site of genome packaging, which may be either be at the nuclear pores, within the nucleus, as suggested by Scheifele et al. [36], or in the cytoplasm juxtaposed to the outer nuclear membrane.

Interestingly, the KKPKR motif resembles a classical nuclear localization signal [43] and we have previously found that over expression of the nuclear pore associated, Ubc9 protein lead to a dramatic redistribution of Pr78 to the nuclear compartment [44]. Experiments are in progress to determine whether the KKPKR motif can function as a nuclear targeting signal when fused to a heterologous protein. If the KKPKR does function as an NLS to cycle Pr78 through the nucleus during the virus life cycle, as has been suggested for RSV, its export to the cytoplasm does not utilize the CRM-1 pathway (Fig. 6B). An alternative hypothesis, which is consistent with the data presented here, is that the KKPKR sequence targets Pr78 to the nuclear pore, where it first recognizes the vRNA during Tap-mediated RNA export [45]. This Gag-vRNA complex would then serve as the nucleation event for spherical capsid assembly just outside of the nuclear pore where the betaretroviruses are known to assemble.

A motif within the M-PMV MA domain called the cytoplasmic targeting/retention signal (CTRS), which is located approximately 100 residues upstream of the KKPKR motif, has also been implicated in directing Pr78 to the intracellular site of assembly. Mutant Pr78 proteins (R55W) that contain an arginine to tryptophan substitution at position 55 in MA do not accumulate at the usual cytoplasmic sites of assembly. Instead R55W-Pr78 proteins are targeted the plasma membrane where they assemble concomitantly with budding, as with the C-type retroviruses [20]. This arginine is contained within an 18 amino acid sequence (residues 43–60) that is conserved between M-PMV and MMTV. When these 18 residues were inserted in the MA domain of C-type MLV Gag, MLV capsid assembly occurred in the cytoplasm [46]. Whether or not these altered MLV capsids assembled in the perinuclear/pericentriolar region of the cytoplasm was not shown. It has, therefore, been suggested that these residues either target Pr78 molecules to the cytoplasmic assembly site or they retain Pr78 at this site until the procapsids are fully assembled.

We hypothesize that the KKPKR motif identified in this study, which may be included in a larger motif that has yet to fully defined, functions either prior to or separate from the CTRS function. We speculate that the KKPKR motif is involved in targeting Pr78 to the nuclear pore to facilitate RNA packaging. Because only two copies of vRNA are packaged into virions, only a few Gag proteins need to be targeted there, which is consistent with the findings present here that the majority of Gag proteins don't associate with nuclear pores. Once Gag has associated with the vRNA, the Gag-vRNA complex would then be transported to the assembly site perhaps via the CTRS signal to initiate spherical capsid assembly.

## Materials and methods

### DNAs

Plasmid pSARM4 is an infectious molecular clone of wild-type M-PMV. Plasmid pMT.ΔE is an *env *deletion mutant of pSARM4 [47]. Deletion of the KKPKR motif was accomplished using the Altered Sites II Mutagenesis System (Promega) as per manufacture's protocol. Briefly, the 1,307 bp, *Sph*I-*Pvu*II fragment (nt 171-1478) of pSARM4 was subcloned into the *Sma*I-*Sph*I sites of pALTER. Mutagenesis was carried out using the mutagenic oligonucleotide (5'-GTTTGTGCTCTTAACAGAACT GGGAAAGTACTTGATAAACCTTTATCTTGTAGAGAGG), to precisely delete amino acids 153 through 157 (KKPKR) in M-PMV Gag. The mutation was subcloned back into pSARM4 using *SacI *and *PacI *sites. After mutagenesis, plasmid DNAs were sequenced to ensure that unwanted mutations were not inadvertently created. Plasmid pETM100A is a prokaryote expression vector used to express a (His)_6_-tagged M-PMV Gag protein in *E. coli *(32). Plasmid pRS.V8-EGFP was used to express a RSV Gag-EGFP fusion protein in mammalian cells (John Wills, Pennsylvania State University College of Medicine) [29].

### Cell lines and transfection

COS-1 and HOS cells were grown at 37°C with 5% CO_2 _in Dulbecco's modified Eagle's medium supplemented with 10% fetal bovine serum. HeLa cells were grown in RPMI 1640 medium supplemented with 10% fetal bovine serum and 5% tryptose phosphate broth. DNA transfections were carried out using Fugene 6 (Roche Diagnostics, Indianapolis, IN) following the manufacture's protocol.

### Antibodies

Goat anti-M-PMV antibodies were obtained from Eric Hunter (Emory University). Mouse monoclonal antibodies that recognize the conserved FG repeats found in nuclear pore complex proteins (MAb414) were purchased from Covance Research Products (Berkely, CA). HRP-conjugated goat anti-rabbit IgG and HRP-conjugated goat anti-mouse IgG were purchased from Amersham Pharmacia Biotech (Little Chalfont Buckinghamshire, England). Cy2 conjugated donkey anti-rabbit and Cy5 conjugated donkey anti-mouse were purchased from Jackson Immunoresearch Laboratories (West Grove, PA). Rabbit polyclonal anti-Pr78 (47) was affinity purified as follows. M-PMV Gag proteins containing a carboxy-terminal (His)_6 _tag were expressed in *E. coli *BL21 (DE3) cells from the expression plamsid pET.M100A for 4 hours in the presence of 0.1 mM IPTG. Cells were harvested by centrifugation at 4,000 × g and lysed at room temperature in a denaturing lysis buffer containing 8 M urea in TNI pH 8.0 buffer (50 mM Tris Cl pH 8.0, 150 mM NaCl and 0.5 mM imidizole). Cellular debris was removed by centrifugation at 10,000 × g and the supernatant was passed over an Ni-NTA agarose nickel column (Qiagen Sciences, Maryland, USA) and extensively washed in 8 M urea in TNI pH 6.5 buffer. The denatured Gag proteins on the column were refolded using a slow (8 h), linear reduction of urea from 8 M to 0 M (in TNI buffer, pH 8.0) in a manner similar to that described by Klikova *et al*. [48] who have shown that M-PMV Gag proteins denatured in 8 M urea could be refolded into a confirmations that are competent to assemble into immature capsids *in vitro *by slowly removing the urea over an 8 hour period at 4°C. After refolding, Gag proteins were eluted form the Ni^2+ ^column using TNI pH 8.0 buffer containing 0.1 mM EDTA. After exchanging the TNI-EDTA buffer to pH7.2 Coupling Buffer (Pierce) by dialysis, the Gag proteins were covalently coupled to activated agarose beads using the Amino-Link Plus kit (Pierce, Rockford, Il) per manufacturer's protocol. Anti-Pr78 antibodies in the immunized rabbit serum were affinity purified using the immobilized Gag column by standard protocols [49].

### Intracellular Gag Fractionation and Immunoblots

Intracellular Gag fractionation experiments were used to assay intracellular procapsid assembly. 60 mm diameter culture dishes containing either untransfected or transfected COS-1 cells were lysed in 1 ml TX-100 lysis buffer (0.25 M sucrose, 1.0 mM EDTA, 10 mM Tris-HCl [pH7.5], 0.14 M NaCl, 0.5% Triton X-100, 0.25% DOC) containing PMSF, leupeptin, pepstatin, and aprotinin for 30 minutes on ice. Cell debris and nuclei were removed by centrifugation for 2 min in a microfuge. The pellets were discarded and the capsid-containing supernatants were fractionated by centrifugation through a 20% sucrose cushion at 350,00 × g in a MLA-130 rotor for 30 minutes at 4°C. The pelleted procapsids were lysed in 2× protein loading buffer (10% glycerol, 2.3% SDS, 63 mM Tris-HCL [pH6.8], 5% β-mercaptoethanol, and 0.01% bromophenol blue). Lysates were boiled for 3 minutes, separated on a 12% SDS PAGE gel, transferred to nitrocellulose. Viral proteins were analyzed by western blot using polyclonal anti-Pr78, HRP-conjugated goat anti-rabbit IgG, and Western Lightning Chemiluminescence Reagent Plus (Perkin-Elmer) as per manufacturers suggestions.

### Radiolabeling and immunoprecipitation

For this, subconfluent 60 mm diameter tissue culture dishes containing either untransfected and transfected COS-1 cells were washed twice in DMEM without methionine or cysteine (DMEM Met^- ^Cys^-^) and starved in 4 ml of DMEM Met^- ^Cys^- ^for 15 min at 37°C in 5% CO_2_. The medium was replaced with 800 μl of DMEM Met^- ^Cys^- ^containing 250 μCi [^35^S] methionine-cysteine (>1000 Ci/mmol; NEN, Boston, MA). The cells were incubated for 30 minutes at 37°C, 5% CO_2_. Cells were either immediately lysed (pulse) or washed with complete medium and then incubated for 1, 2, 3, 4, or 8 hours in complete medium (chase) prior to lysis.

Virion and cell lysis were lysed as follows. Culture medium from each plate was collected and clarified by centrifugation in a microfuge for 2 min at 13,000 rpms. Virions were lysed by adjusting the culture medium to 1× lysis buffer B by adding 1/5 volume of 5× lysis buffer B (0.5% sodium dodecyl sulfate [SDS], 5% Triton X-100, 5% deoxycholate [DOC], 0.75 M NaCl, 0.25 M Tris-HCl [pH 6.8]). Cell monolayers were lysed in 1 ml lysis buffer A (Triton X-100, 1% DOC, 0.15 M NaCl, 0.05 M Tris-HCl [pH 6.8]) containing PMSF, leupeptin, pepstatin, and aprotinin for 30 minutes on ice. Cell debris and nuclei were removed by centrifugation for 2 min in a microfuge. The pellets were discarded and the capsid-containing supernatants were adjusted to 1× lysis buffer B by adding 0.1% SDS. All lysates were precleared by incubation with inactivated, formalin-fixed *Staphylococcus aureus *cells. Viral proteins were immunoprecipitated from all samples using Rbt anti-Pr78 antibodies and *Staphylococcus aureus *cells as previously described [50]. Immunoprecipitates were resuspended in protein loading buffer (10% glycerol, 2.3% SDS, 63 mM Tris-HCL [pH6.8], 5% β-mercaptoethanol, and 0.01% bromophenol blue), boiled for 3 minutes, separated by SDS PAGE gel (12% acrylamide), and visualized by fluorography or analyzed by phosphor-imaging using The Discovery Series Quantity One (BioRad, Hercules, CA).

Steady state radiolabeling with [^3^H] leucine was done to assess glycoprotein incorporation and Pr78 processing. Transfected COS-1 cells were starved in leucine-free DMEM for 90 minutes, and labeled overnight with [^3^H] leucine (500 μCi/ml, 173.0 Ci/mmol, NEN, Boston, MA). The culture medium was filtered with a 0.45 μm syringe filter, and [^3^H] leucine-labeled viruses were pelleted through a 25% sucrose cushion by centrifugation at 350,000 × g in a TLA 100.3 rotor for 30 minutes at 4°C. After the viral pellet was solublized in 1× lysis buffer B, the viral proteins were using goat anti-M-PMV antibodies and analyzed as described above.

### Virus replication assay

The infectivity of wild-type and mutant viruses were determined by measuring the increase of reverse transcriptase (RT) activity in the culture supernatants of inoculated HOS cell cultures at various times postinfection. Culture medium was harvested from COS-1 cells that had been transfected 48 h previously with either wild-type or mutant DNA. Loose cells and cellular debris were pelleted by centrifugation for 2 min in a microfuge and the level of RT activity in the clarified culture medium was measured. Hos cells were infected with equivalent amount of RT-containing in the presence of 4.0 μg/ml of polybrene. Culture fluids were harvested at various days postinfection and assayed for RT activity. RT assays were carried out by pelleting virus from medium by centrifugation at 350,000 × g in a TLA 100.3 rotor for 30 minutes at 4°C. The viral pellet was lysed in 12 μl of Virion Lysis Buffer (50 mM Tris-HCl [pH 7.8], 100 mM KCl, 0.05% TX-100, 2 mM diothiothreitol [dTT]) on ice for 15 min. 7.5 μl of virion lysates were added to 30 μl of RT Reaction Buffer (50 mM Tris [pH 8.0], 100 mM KCl, 2 mM dTT, 7.5 mM MgCl_2_) with 8 μCi [^32^P] α-TTP (>1000 mCi/mmol, NEN) and 1.25 μg poly (A) – oligo (dT)_15 _(Roche). The RT reaction was incubated at 37°C for 2 hours. 10 μl of the RT reaction was spotted onto a piece of Whatman DE81 paper and allowed to dry. The filter paper was washed twice for 15 min in 2× SSC buffer (0.3 M NaCl, 0.03 M Na Citrate), twice briefly in 95% EtOH, and once in distilled water. The filters were dried and [^32^P] incorporation was measured by scintillation counting.

### RNA extraction and RT-PCR

Medium from transfected COS-1 cells were clarified and virus was pelleted through a 20% sucrose cushion at 207,570 × g in an SW41 rotor for 2 hours at 4°C and resuspended in 30 μl of PBS. The amount of viral particles was normalized by quantitation of p27 detected by immunobloting. RNA was extracted from equivalent amounts of virus using QIAamp Viral RNA Mini Kit (Qiagen Sciences, Maryland, USA) as per manufacturers suggestions. Purified RNA was treated with 1 U of Rnase-free Dnase I (New England Biolabs, Inc., Berverly, MA) for 30 min at 37°C, followed by inactivation at 70°C for 30 min. Purified RNA from equivalent amounts of virus was diluted 1:1,000 followed by 2-fold serial dilutions. 5 μl of diluted RNA was used for first-strand cDNA synthesis as per manufacturers suggestions for M-MLV RT (Invitrogen) using 500 ng of oligo (dT)^12–18 ^(Ambion, Austin, TX). First-strand cDNA (5 μl) were amplified by PCR using the following oligos 5'-CCGCTCGAGCGGGCCGCCATGCCGGTGGCTGAAACCGTTG and 5'-GCTCTAGAGCGGCGGCCATGGCCAGG to amplify M-PMV CA sequences. The relative amount of viral RNA packaged into viral particles was estimated by end-point dilution.

### Electron microscopy

Transmission Electron Microscopy (TEM) was utilized to view assembled intracellular procapsids. Transfected COS-1 cells were fixed for 1 hour with two changes of 3% glutaraldehyde in 0.1 M phosphate buffer (5 mM NaH_2_PO_4 _and 5 mM phosphoric acid). The cells were rinsed for 30 min with 0.1 M phosphate buffer, followed by osmication (2% OsO4 in phosphate buffer) for 1 hour. The cultures were washed and dehydrated with a graded series of ethanol (25% 50%, 75%, 95% 100), followed by a graded series of ethanol/Epon 812 (Shell) mixtures (3:1, 1:1, 1:3). The cells were infiltrated with pure Epon 812 and polymerized at 60°C for 48 hours. Thin sections were made with a LBK Ultrotome III, mounted on copper grids, and stained with 2% uranyl acetate and lead citrate. Thin sections were examined and photographed with a HitachiH-7500 transmission electron microscope operated at 60 Kv.

### Subcellular localization

The intracellular localizations of Pr78^Gag ^and Pr78^ΔKR ^proteins were determined by confocal microscopy. Transfected HeLa cells were grown on sterile coverslips in 35 mm culture dishes. At 48 hours post transfection, cells were washed with PBS (137 mM NaCl_2_, 2.7 mM KCl_2_, and 8 mM Na_2_HPO_4_, 2 mM KH_2_PO_4_), fixed in either 4% paraformaldyhyde in PBS for 20 minutes at room-temperature and then subsequently permiabilized with 0.2% TX-100 in PBS for 5 minutes at room-temperature, or fixed in 100% methanol at -20°C for 10 minutes. The coverslips were washed with PBS, and blocked with Blocking Buffer 1 (PBS, 0.2% tween 20, 0.4% fish skin gelatin [Sigma]) for 5 minutes, and then blocked with Blocking Buffer 2 (PBS, 0.2% tween 20, 2.5% goat serum [Sigma]) for 5 min. Affinity purified anti-Pr78 and MAb414 (in PBS, 0.2% tween 20, 2.5% goat serum) were incubated on the coverslips for 45 min at 37°C. The primary antibodies were removed and the coverslips were blocked as they had been previously. Cy2 and Cy5 conjugated secondary antibodies (in PBS, 0.2% tween 20, 2.5% goat serum) were incubated on the cover-slips for 30 min at 37°C and washed in PBS with 0.2% tween 20. The coverslips were mounted on slides using GEL-mount and analyzed by confocal microscopy (Olympus FV500 w/upright BX Olympus florescence microscope). 0.3 μm Z-sections were stacked and orthogonal views through the cell were generated using Flowview imaging analysis software (Olympus).
